# Social and Poor Law Problems

**Published:** 1908-04-04

**Authors:** 


### SOCIAL AND POOR-LAW PROBLEMS.

### SANITARY ADMINISTRATION AND SMALL PROPERTY OWNERS.

Occasionally one sees in the newspapers advertisements of property which promises to return a remarkably high rate of interest, the said interest being worked up by leaving the greater part of the purchase money on mortgage. This is the sort of thing that attracts the small investor, who needs more than an ordinary return on his money to make anything approaching an income out of it. The property thus advertised is almost invariably in a poor locality, and it is sure to be in bad condition. As a rule the vendor is a man who has taken the best out of it, and sells it when it is likely to need a good deal in the way of repairs. He therefore finds it more profitable to become the mortgagee, leaving some other to bear the burden of the property. If there are signs of the sanitary authorities becoming more active than they have been, that is an additional reason for getting rid of the houses. And one who watches the signs of the times can tell when such a thing is likely to happen. For the Local Government Board has its eyes on the mortality rates of the country, and if in any district these rise suddenly, they direct an inspector to make inquiry into the reason of it. If, for example, a large proportion of the deaths occur in a neighbourhood where midden privies are the rule, orders may be given for the immediate provision of proper drainage. The cost of this would devour all the profits of the property, and therefore there will always be an outcry against it on the part of the small owner, who is depending on this for part, or, it may be, the whole, of his living. It is for this reason that lone women, who have lived happily on the rental of property which was, perhaps, built by their parents, feel themselves robbed by the authorities of their town and village when they get an order to execute repairs on their property which, to an outsider, seem absolutely necessary. But the lone woman grumbles and submits. When it is a small tradesman who has put his savings into this sort of insanitary property the case is different; for he may be himself on the parish or borough council, or at least have some influence there, and thus may steadily hinder the course of sanitary improvement. He is the more likely to achieve this if the Medical Officer of Health for the district is, as so many are, only partly employed by the council, and ekes out his living by private practice. It is a brave man who will offend a good private patient by speaking ill of the conditions of life in houses which that patient owns, and no medical man ought to be put into such a position. For in many cases no ordinary repairs will make the houses sanitary, and to order their abandonment or their demolition and reconstruction seems to the man who depends on such houses alone for his living to be sheer robbery. Here the large landowner is better to deal with. He has probably inherited his property, and therewith the sense of responsibility for his tenants' well-being. At least he knows that property has responsibilities as well as privileges; or, if he has purchased it, he is the more likely to take a pride in having the houses he owns as good as or better than his neighbour's. But the man who must starve if his property is to be improved is, quite comprehensibly, the worst foe to betterment. When, as not infrequently happens, the majority of a council are of this class, it becomes necessary, in the interests of the community as a whole, for the central authority to override the wishes of the local one. It is an ungracious task, but the records of many rural districts show that in the matter of sanitation the local authorities cannot be left to act—or refuse to act—as they please.

